# Assertive Community Treatment For People With Alcohol Dependence: A Pilot Randomized Controlled Trial

**DOI:** 10.1093/alcalc/agw091

**Published:** 2016-12-09

**Authors:** Colin Drummond, Helen Gilburt, Tom Burns, Alex Copello, Michael Crawford, Ed Day, Paolo Deluca, Christine Godfrey, Steve Parrott, Abigail Rose, Julia Sinclair, Simon Coulton

**Affiliations:** 1 Addictions Department, National Addiction Centre, Institute of Psychiatry, Psychology and Neuroscience, King's College London, 4 Windsor Walk, LondonSE5 8BB, UK; 2 King's Fund, 11 Cavendish Square, LondonW1G 0AN, UK; 3 Department of Psychiatry, Warneford Hospital, University of Oxford, OxfordOX3 7JZ, UK; 4 School of Psychology, University of Birmingham, Edgbaston, BirminghamB15 2TT, UK; 5 Center for Mental Health, Imperial College London, Commonwealth Building, Hammersmith Campus, Du Cane Road, LondonW12 0NN, UK; 6 Department of Health Sciences, University of York, Seebohm Rowntree Building, University of York, Heslington, YorkYO10 5DD, UK; 7 Psychological Sciences, Institute of Psychology, Health and Society, University of Liverpool, Waterhouse Building, LiverpoolL69 3BX, UK; 8 Clinical and Experimental Sciences, Faculty of Medicine, University of Southampton, University Road, SouthamptonSO17 1BJ, UK; 9 Centre for Health Services Studies, George Allen Wing, Cornwallis Building, University of Kent, Canterbury, KentCT2 7NF, UK

## Abstract

**Aims:**

A pilot randomized controlled trial (RCT) to assess the feasibility and potential efficacy of assertive community treatment (ACT) in adults with alcohol dependence.

**Methods:**

Single blind, individually randomized, pilot RCT of 12 months of ACT plus treatment as usual (TAU) versus TAU alone in adults (age 18+ years) with alcohol dependence and a history of previous unsuccessful alcohol treatment attending specialist community alcohol treatment services. ACT aimed to actively engage participants for 12 months with assertive, regular, minimum weekly contact. ACT was combined with TAU. TAU comprised access to the full range of services provided by the community teams. Primary outcome is mean drinks per drinking day and percent days abstinent at 12 months follow up. Analysis of covariance was conducted using 80% confidence intervals, appropriate in the context of a pilot trial.

**Results:**

A total of 94 participants were randomized, 45 in ACT and 49 in TAU. Follow-up was achieved with 98 and 88%, respectively at 12 months. Those in ACT had better treatment engagement, and were more often seen in their homes or local community than TAU participants. At 12 months the ACT group had more problems related to drinking and lower quality of life than TAU but no differences in drinking measures. The ACT group had a higher percentage of days abstinent but lower quality of life at 6 months. The ACT group had less unplanned healthcare use than TAU.

**Conclusions:**

An trial of ACT was feasible to implement in an alcohol dependent treatment population.

**Trial registration:**

ISRCTN22775534

## Introduction

Excessive alcohol use places a considerable burden on society, and is the fifth leading cause of disability globally (Lim *et al*., 2012). In England, ~4% of the adult population are alcohol dependent (McManus *et al*., 2009) and the government has identified provision of effective treatment as a priority in reducing these costs (HM Government, 2012). Current specialist alcohol service provision in the UK focuses on discrete, time-limited episodes of intervention, typically up to 12 weeks duration and emphasizes personal choice and motivation. However, there are high rates of non-attendance in the treatment population (Mitchell and Selmes, 2007), <40% complete a treatment programme (Passetti *et al*., 2008), and after successful completion approximately one-third continue to drink heavily and have poor long-term outcomes (Marshall *et al*., 1994). For this group of patients alcohol dependence is a chronic relapsing disorder and in typical practice, treatment episodes extending over many years is typical. Current alcohol care pathways require significant levels of motivation and self-efficacy to navigate, that few patients possess (Gilburt *et al*., 2015).

Assertive community treatment (ACT) is a model of intensive case management which has been shown to be effective in improving engagement and retaining those with serious mental illness and those with alcohol and drug use comorbidities (Dieterich *et al*., 2010). A number of studies have applied individual components of ACT to the treatment of alcohol dependence with positive outcomes (Gilbert, 1998; Stout *et al*., 1999; Hilton *et al*., 2001). A non-randomized cohort study of assertive engagement methods resulted in a significantly greater number of patients completing treatment and entering aftercare (Passetti *et al*., 2008). However, the potential benefits of individual elements of ACT in patients with alcohol dependence without severe mental illness remain unclear, and there have been no published randomized controlled trials investigating the impact of ACT in this population to date. We therefore conducted a pilot randomized controlled trial of ACT in people with alcohol dependence and a history of previous unsuccessful treatment to investigate (a) the feasibility of recruiting and retaining people seeking treatment for alcohol dependence in a clinical trial of ACT and (b) the potential efficacy of ACT on the drinking behaviours and quality of life of people with alcohol dependence and a history of disengagement from specialist alcohol treatment services.

## Method

This study (ACTAD trial) was reviewed and approved by the National Research Ethics Service Committee London—Chelsea (REC number: 08/H0801/113) and the trial was registered with the International Standard Randomized Controlled Trial registry (ISRCTN22775534) prior to the commencement of data collection. Progress of the trial, adherence to protocol and participant safety were overseen by an independent trial steering committee. A detailed protocol for the study has already been published (Gilburt *et al*., 2012).

### Trial design

We undertook a single blind, pilot randomized controlled trial of ACT plus treatment as usual (TAU) compared with a TAU alone for people with alcohol dependence. The trial was conducted across two NHS trusts in South London: participants were recruited at the point of referral to one of three specialist community drug and alcohol services for a new episode of treatment. Participants were recruited between 23 March 2010 and 23 January 2012.

An equal number was randomized to each treatment condition. Randomization was conducted using a secure independent service at the level of the individual and was stratified by alcohol dependence (≤30 and >30) as measured by the Severity of Alcohol Dependence Questionnaire (Stockwell *et al*., 1979) and by site.

As a pilot study, the sample size was selected to determine feasibility and to allow for an analysis of potential effect prior to designing a full scale definitive trial. In order to ascertain these parameters, we considered a clinically meaningful difference in alcohol consumption between the groups at 12 months to be of the order of 30% and estimated the numbers required with 80% power and an alpha of 0.2, appropriate for a pilot study, using two-sided test. In addition, we considered an acceptable rate of follow-up at 12 months to be not less than 75%. Our required sample size was estimated as 45 in each group at baseline, with the expectation that at least 34 would be followed-up at 12 months.

### Recruitment

All potential participants were in the first instance identified and approached by a member of the community drug and alcohol team who informed them about the trial and obtained verbal consent to being contacted by a researcher. A member of the research team met with the participant to explain the trial further and check eligibility for the trial. Participants who met all the criteria were invited to provide written consent for the trial.

### Inclusion and exclusion criteria

All participants met the following criteria: (a) age 18 years or over; (b) able to understand English sufficiently well to obtain informed consent and complete the assessment instruments; (c) attended an NHS community addiction service in either of the participating trusts for alcohol dependence on at least one previous occasion in the last 5 years; and (d) an ICD-10 diagnosis of alcohol dependence as determined using the Composite International Diagnostic Interview (Robins *et al*., 1988). We excluded patients who were: (a) unable to provide written informed consent; (b) street homeless; (c) diagnosed with a psychotic disorder; (d) in receipt of assertive outreach services or had community mental health team input once a month or more; (e) had severe cognitive impairment as determined by the Mini Mental State Examination score of ≤10 (Folstein *et al*., 1975); and (f) who had a history of violence to staff or were registered under the UK Multi-Agency Public Protection Arrangement.

### Interventions

#### Assertive community treatment

The intervention was informed by the original ACT model used for people with psychosis and findings from research identifying effective elements of assertive outreach in UK studies (Burns *et al*., 2000). The ACT intervention comprised:

- A maximum caseload of 15 ACT patients per ACT practitioner.
- Input from a multidisciplinary team (including psychiatrists and substance misuse specialists).
- Regular contact (minimum of once a week), with 50% of contacts occurring outside of the service settings either in the patients’ home or neighbourhood, and in which short frequent contacts rather than long complex contacts were encouraged.
- Assertive engagement where there were persistent and repeated attempts to contact, and an emphasis on maintaining contact and building relationships.
- A focus on both health and social care needs, including accommodation, leisure, occupation and physical and mental health.
- A flexible approach, focusing on the patient's goals even when these were peripheral to the alcohol dependence.
- Practitioners were explicit about their role both in care planning and in visits.
- An ethos of ‘going out of your way’, where practitioners are encouraged to step outside of professional roles and ‘go the extra mile’ for patients.
- Extended care provided for a prolonged period of 1 year.

An intervention manual was developed in collaboration with experts in the provision of ACT. The manual outlined the core components of the ACT intervention and the principles of delivery in line with components of the alcohol treatment pathway. This included hours of operation, caseload, target client group, source of referrals, assessment, interventions and care plan and the use of interventions for alcohol dependence. Policies on training and development for ACT practitioners, home visiting, lone working and risk assessment and the withdrawal from treatment were also incorporated. Finally the manual provided an overview of the research processes involved in the trial.

#### Treatment as usual

Participants randomized to the intervention arm received TAU plus ACT, while those randomized to the control group received TAU alone. TAU included the allocation of a keyworker when available with contact as required as per local team policies. Contact was primarily conducted within a service setting (community addictions service or general practice) via an appointment-based system. These NHS services specialize in treatment of patients with drug and/or alcohol dependence. They are located in community-based treatment centres and staffed by multidisciplinary teams comprising specialist addiction psychiatrists, nurses, clinical psychologists, social workers, counsellors and community support workers. They provide a wide range of interventions in accordance with national clinical guidelines (Department of Health, 2007; National Institute for Health and Care Excellence, 2011). Participants received a full assessment of alcohol, social and physical health needs and a risk assessment. The focus of treatment was primarily on alcohol dependence, promoting abstinence and relapse prevention. This included access to medical detoxification, psychological interventions focused on drinking behaviour and aftercare as required. TAU also included input from specialists in addiction psychiatry, clinical psychology and social work where available. When these services were not directly provided by the community drug and alcohol treatment service, participants were most often referred or signposted to other relevant agencies as required. The majority of participants were discharged to primary care from the specialist service within 12 weeks of being allocated a keyworker unless significant risks were identified. Failure to attend several appointments resulted in discharge from the service. These approaches are broadly in line with national service delivery at the time of the study. In addition we collected data on the actual care received in order to understand the content of TAU.

### Training and support

A training programme for ACT staff was developed by the team in collaboration with experts in ACT and addictions treatment. The training comprised workshops providing information on ACT, its history, implementation in mental health and trial processes; a 1-day clinical placement with a mental health assertive outreach team shadowing a member of staff; and a final workshop day focused on the application of ACT in addictions services. A total of seven practitioners from the trial sites attended the training (four from one service, three from the second) including substance misuse, nursing and addiction psychiatry professionals. Additional training was delivered on a one-to-one basis to a further three practitioners at a third service setting following late incorporation into the trial, and in response to staff turnover.

In addition to existing team-based clinical supervision, practitioners delivering ACT were encouraged to attend monthly ACT group supervision meetings throughout the course of the study. These meetings were facilitated by the Chief Investigator, an experienced addictions psychiatrist, and the trial manager. Meetings focused on enabling practitioners to share experience and practice in order to support delivery of ACT, address clinical issues arising as a result of the intervention, and reinforce fidelity of the ACT intervention.

### Intervention fidelity

Staff providing care to participants in both arms of the trial completed a contact log detailing the care they provided for each patient following each contact. The log, developed from a previous study of assertive outreach, included details about the mode of contact, (i.e. face to face, telephone), setting, focus of contact, and the member of staff involved (Burns *et al*., 2000). To enhance accuracy, data were additionally collected from the electronic clinical records of each participating service following completion of the trial. Data highlighting additional contacts with a participant and information absent from contacts recorded in the log was used to supplement existing information.

### Outcome measures

Feasibility and acceptability of delivering ACT to people with alcohol dependence was measured by levels of recruitment into the trial and retention of participants in both arms of the study at 12 months follow-up.

The primary outcome measures were mean drinks per drinking day and percent days abstinent at 12 months measured using the Time Line Follow Back form 90 (TLFB; Miller, 1995). Secondary outcomes included total alcohol consumed, other consumption measures at 6 months and other drug use measured using TLFB, alcohol-related problems (Alcohol Problems Questionnaire; APQ; Drummond, 1990), severity of alcohol dependence (Severity of Alcohol Dependence Questionnaire; SADQ; Stockwell *et al*., 1979), health utility (EQ-5D; EuroQol Group, 1990), health-related quality of life (SF-12; Ware *et al*., 1996), motivation to change (Readiness to Change Treatment version; Heather *et al*., 1999), social network involvement (Important People and Activities Inventory; Zywiak *et al*., 2002), health service utilization (York Service Use Questionnaire; Drummond *et al*., 2009). All measured at baseline and then 6 and 12 months after randomization. Data were collected through face-to-face interviews conducted by trained researchers working on this trial who were blinded to the treatment allocation. All participants were reminded by the researcher at the beginning of each follow-up interview not to reveal the treatment allocation they received. For each time point (including the baseline) assessment was made was of the previous 6 months. However, in the case of TLFB form 90 for alcohol and drugs the time period covered at each time point was the previous 90 days.

### Statistical analysis

The main hypothesis, stated as a null hypothesis, is that TAU augmented with ACT is no more effective than TAU in reducing alcohol consumption 12 months after randomization. The primary outcome measure was mean drinks per drinking day and percent days abstinent at 12 months follow up. Our primary analysis was by intention-to-treat in which participants are analysed as part of their allocated group irrespective of the treatment received. This provides the most rigorous estimate of effectiveness. The primary outcome measure was analysed using an analysis of covariance approach adjusting for known confounding variables such as baseline mean drinks per drinking day, age and gender. If the assumptions underlying ANCOVA were not met transformations were undertaken and if transformations were not viable alternative non-parametric approaches were used. Continuous secondary measures were analysed in a similar manner. Categorical variables were analysed using chi-squared statistics and binary outcomes analysed using logistic regression controlling for known confounding variables. Estimates and the 80% confidence intervals are presented.

## Results

### Feasibility of recruitment

**Fig. 1.**
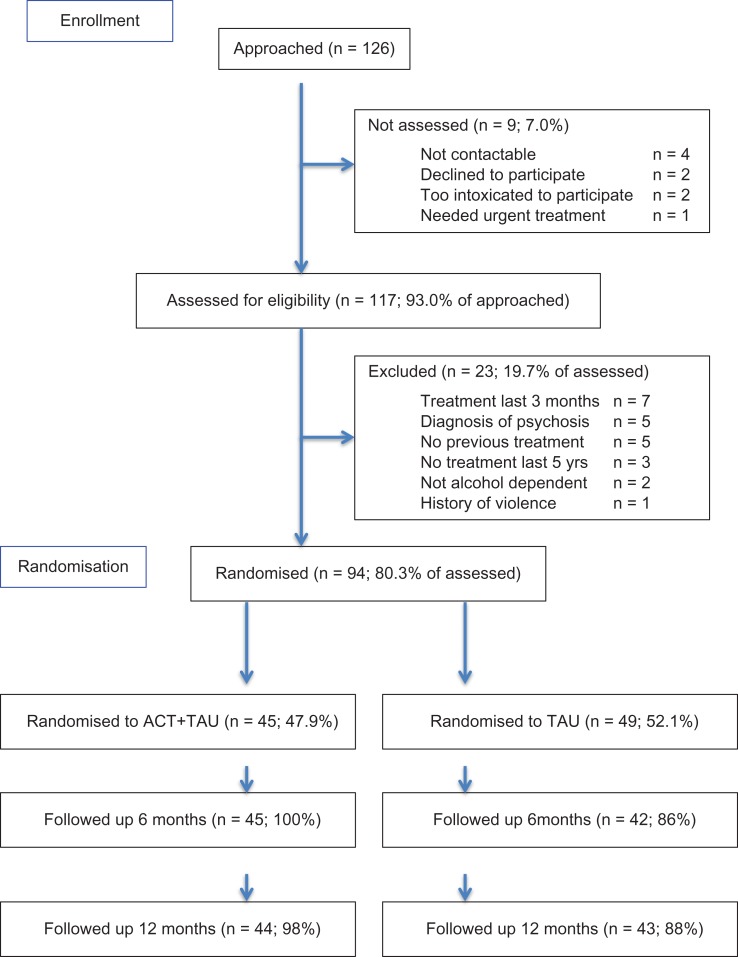
Trial consort diagram.

Feasibility outcomes for this study were assessed by recruitment and retention rates in both arms of the study. Participant flow is indicated in the Consort diagram (Fig. 1). Of 126 individuals assessed for eligibility, four could not be contacted after referral by the team, two declined to participate, two were assessed as too intoxicated to participate and one needed to be seen immediately by the clinical team so could not be seen by a researcher. A total of 117 (93.0%) patients were assessed for eligibility to take part. Of all, 23 did not meet the inclusion criteria (seven had been seen for treatment in the last 3 months, five had been diagnosed with a psychotic disorder, five had no history of treatment with the service, three had not been seen by the service within the last 5 years, two were not alcohol dependent and one had a significant history of violence).

A total of 94 (80.3%) provided consent and were randomized. Overall, 45 participants were randomized to ACT plus TAU arm (47.9%) and 49 participants were randomized to the TAU arm (52.1%). The follow-up rates at 6 months were 100% (*n* = 45) in the ACT plus TAU arm and 86% (*n* = 42) in the TAU arm. The follow-up rates at 12 months were 98% (*n* = 44) in the ACT plus TAU arm and 88% (*n* = 43) in the TAU arm.

### Baseline characteristics

The baseline demographics of the sample are shown in Table 1. The majority of the sample was male (61%), white (87%), not currently in a marital relationship (68%), and living in rented or temporary accommodation (76%), were parents (68%), and had a basic level of educational qualifications (67%). The mean age was 43 years.

**Table 1.** Demographics of the sample

	ACT + TAU (*n* = 45)	TAU (*n* = 49)	Overall (*n* = 94)
Mean age (SD)	42.6 (9.6)	43.2 (9.3)	43.0 (9.6)
Male, *n* (%)	26 (58)	31 (63)	57 (61)
Ethnicity, *n* (%)			
White	38 (84)	44 (90)	82 (87)
Black	2 (4)	1 (2)	3 (3)
Asian	3 (6)	2 (4)	5 (4)
Other	4 (2)	2 (4)	6 (6)
Marital status, *n* (%)			
Never married	18 (40)	20 (41)	38 (40)
With partner	6 (13)	4 (8)	10 (11)
Married	10 (22)	10 (20)	20 (21)
Separated	6 (13)	5 (10)	11 (12)
Divorced	5 (12)	8 (16)	13 (14)
Widowed	0	2 (5)	2 (2)
Accommodation, *n* (%)			
Owner/ occupier	11 (24)	12 (24)	23 (24)
Rented/ temporary	34 (76)	37 (76)	71 (76)
Educational qualifications, *n* (%)			
None	17 (38)	14 (28)	31 (33)
GCSE or equivalent or higher	28 (62)	35 (86)	63 (67)
Children, *n* (%)	30 (67)	34 (69)	64 (68)

At baseline no significant differences were observed between the groups in terms of study outcome measures (Table 2). All participants met the criteria for alcohol dependence with lower than population average mental and physical health-related quality of life. The majority were in the pre-contemplative stage of change and had high levels of alcohol dependence.

**Table 2.** Outcomes by allocated group at baseline, 6 and 12 months

	Baseline		Month 6		Month 12	
	ACT + TAU (*n* = 45)	TAU (*n* = 49)	ACT + TAU (*n* = 43)	TAU (*n* = 44)	ACT+TAU (*n* = 42)	TAU (*n* = 45)
Alcohol use in past 90 days						
Mean drinks^a^ consumed (SE)	2074 (232.9)	1926 (175.1)	722.8 (167.9)	816.3 (160.6)	728.9 (152.3)	774.1 (148.7)
Mean drinks per day (SE)	23.05 (2.6)	21.41 (1.9)	8.032 (1.9)	9.070 (1.8)	8.099 (1.7)	8.601 (1.6)
Mean percent days abstinent (SE)	13.80 (2.9)	14.24 (2.8)	67.83 (5.8)	57.40 (6.2)	65.45 (5.6)	57.41 (6.0)
Alcohol problems (APQ scored 1 to 23, 23 most severe)	11.48 (0.6)	11.42 (0.5)	6.37 (0.9)	6.02 (0.7)	6.83 (0.9)	5.29 (0.7)
EQ5D (scored 0–1, 0 worse health utility)	0.62 (0.1)	0.50 (0.1)	0.61 (0.1)	0.68 (0.1)	0.61 (0.1)	0.71 (0.1)
Health-related quality of life (SF12 scored 0–100, 0 most severe)						
Mental component	30.61 (1.82)	30.66 (1.93)	37.08 (2.61)	39.15 (2.16)	39.28 (0.06)	40.84 (2.23)
Physical component	42.30 (1.93)	31.50 (1.87)	44.09 (2.07)	44.22 (1.96)	43.93 (1.99)	45.58 (1.99)
Readiness to change, *n* (%)^b^						
Pre-contemplation	0	0	1 (3.3)	1 (2.9)	1 (4.8)	0
Contemplation	35 (81.4)	37 (75.5)	15 (50)	16 (48.4)	14 (66.7)	21 (77.8)
Action	8 (18.6)	12 (24.5)	14 (46.7)	17 (50.0)	6 (28.6)	6 (22.2)
Severity of dependence (SADQ scored 0–60, 60 most dependent)	32.38 (2.102)	31.86 (2.043)	18.19 (2.233)	20.41 (2.581)	18.50 (2.273)	15.13 (1.937)

^a^One drink = 1 UK unit of alcohol = 8 g ethanol.

^b^Data on Readiness to Change was not obtained from two participants at baseline and 23 at 6 months, and 39 at 12 months. This was because these Readiness to Change questions were not applicable to participants who were already abstinent at these time points.

### Intervention delivery

Compared with participants randomized to the TAU arm of the trial, participants receiving ACT + TAU were in contact with services for a significantly longer period of time, (*t*(76.77) = 15.62, *P* < 0.001), received a greater mean number of contacts during treatment (*t*(57.75) = 10.52, *P* < 0.001), and received a significantly greater percentage of contacts in a venue other than the addictions treatment service (*t*(66.34) = 7.47, *P* < 0.001).

### Primary outcomes

Tables 3 and 4 contain outcomes at 6 and 12 months. Data presented has been adjusted by baseline covariates; baseline value, age and gender. There were reductions in the total alcohol consumed and increases in the percent days abstinent and number of participants abstinent at 12 months. There were reductions in drinks per drinking day in both groups at 12 months compared to baseline but the difference between allocated groups was small and not statistically significant (Table 5).

**Table 3.** Contact with staff during treatment by allocated group

	ACT + TAU (*n* = 45)	TAU (*n* = 46)	Mean difference (80% CI)	*P*-value
Length of contact, mean days (SD)	335.09 (55.87)	95.54 (87.35)	239.55 (219.72; 259.37)	0.000
Number of contacts with participant, mean (SD)				
Face to face	22.04 (12.79)	5.09 (4.52)	16.96 (14.34; 19.58)	0.000
Telephone	22.47 (12.14)	3.17 (4.29)	19.29 (16.81; 21.78)	0.000
Group	1.98 (4.59)	1.20 (2.78)	0.78 (−0.25; 1.81)	0.330
Number of contacts with carer, mean (SD)				
Face to face	0.16 (0.52)	0	0.16 (0.06; 0.26)	0.051
Telephone	2.50 (4.53)	0.57 (1.38)	1.94 (1.01; 2.86)	0.009
Frequency of contact per week, mean (SD)				
All contacts	1.0 (0.41)	1.90 (2.28)	−0.89 (−1.34; −0.45)	0.012
All contacts with participant	0.95 (0.39)	1.83 (2.30)	−0.88 (−1.33; −0.43)	0.014
Face to face and group contacts	0.48 (0.28)	0.99 (1.71)	−0.51 (−0.84; −0.18)	0.051
Place of contact, mean % (SD)				
Addictions service	20.67 (15.01)	59.49 (31.95)	−38.83 (−45.65; −32.00)	0.000
Home/community	17.84 (17.91)	0.58 (2.67)	17.25 (13.75; 20.76)	0.000
Telephone	54.09 (17.89)	37.49 (32.28)	16.59 (9.54; 23.65)	0.003
Other	7.34 (8.05)	1.86 (7.70)	5.49 (3.35; 7.62)	0.001

**Table 4.** Mean and mean difference of ACT plus TAU versus TAU in outcomes at 6 months adjusted by baseline covariates

	ACT + TAU mean (80% CI)	TAU Mean (80% CI)	Mean difference (80% CI)	*P-*value
Alcohol use in past 90 days				
Mean drinks^a^ consumed	719 (504; 934)	820 (607; 1032)	−101 (−404; 202)	0.67
Mean drinks per day	7.95 (5.57; 10.33)	9.15 (6.79; 11.50)	−1.97 (−4.55; 2.16)	0.64
Mean percent days abstinent	68.43 (60.63; 76.23)	56.81 (49.09; 64.52)	11.62 (0.64; 22.61)	0.18
Number abstinent (%)	14 (32.6%)	13 (29.5%)	–	0.47
Alcohol problems (APQ)	6.37 (5.43; 7.30)	6.03 (5.11; 6.95)	0.34 (−0.97; 1.65)	0.74
EQ5D	0.58 (0.53; 0.64)	0.71 (0.65; 0.76)	−0.12 (−0.21; -0.04)	0.05
Health-related quality of life (SF12)				
Mental component	36.84 (33.90; 39.77)	38.89 (35.99; 41.78)	−2.05 (−6.17; 2.08)	0.52
Physical component	43.73 (41.40; 46.06)	44.33 (42.03; 46.23)	−0.60 (−3.87; 2.68)	0.82
Readiness to change n (%)				
Pre-contemplation	1 (3.3)	1 (2.9)	–	0.96
Contemplation	15 (50.0)	16 (47.1)	–	0.37
Action	14 (46.7)	17 (50.0)	–	
Severity of dependence (SADQ)	17.94 (15.15; 20.74)	20.64 (17.94; 23.34)	−2.70 (−6.59; 1.19)	

^a^One drink = 1 UK unit of alcohol = 8 g ethanol

**Table 5.** Mean and mean difference of ACT plus TAU versus TAU in outcomes at 12 months adjusted by baseline covariates

	ACT+TAU mean (80% CI)	TAU mean (80% CI)	Mean difference (80% CI)	*P*-value
Alcohol use in past 90 days				
Mean drinks^a^ consumed	722 (525; 918)	780 (591; 969)	−58 (−330; 214)	0.78
Mean drinks per day	8.02 (5.85; 10.19)	8.67 (6.58; 10.77)	−0.65 (−3.68; 2.37)	0.78
Mean percent days abstinent	65.56 (57.90; 73.22)	57.30 (49.90; 64.70)	8.26 (−2.39; 18.92)	0.32
Number abstinent (%)	11 (26.2)	13 (28.9)	–	0.48
Alcohol problems (APQ)	6.85 (5.87; 7.83)	5.28 (4.33; 6.22)	1.57 (0.21; 2.93)	0.14
EQ5D	0.59 (0.53; 0.65)	0.72 (0.67; 0.78)	−0.14 (−0.22; -0.05)	0.05
Health-related quality of life (SF12)				
Mental component	38.80 (36.08; 41.53)	41.18 (38.52; 43.85)	−2.05 (−6.17; 2.08)	0.42
Physical component	43.52 (41.10; 45.94)	46.58 (44.21; 48.94)	−2.38 (−6.19; 1.43)	0.25
Readiness to change, *n* (%)				
Pre-contemplation	1 (3.3)	0	–	0.43
Contemplation	14 (66.7)	21(77.8)	–	0.26
Action	6 (28.6)	6 (22.2)	–	
Severity of dependence (SADQ)	18.42 (15.77; 21.07)	15.21 (12.65; 17.78)	3.21 (−0.48; 6.89)	

^a^One drink = 1 UK unit of alcohol = 8 g ethanol.

### Secondary outcomes

Significant differences were observed between the groups at 6 months with the ACT plus TAU group having a higher percentage of days abstinent. At 6 months the TAU group had significantly fewer alcohol-related problems and health utility, measured using the EQ5D, was significantly better for the TAU group at 6 and 12 months. No other significant differences between groups on other secondary outcome measures, including APQ, SF12, Readiness to Change or SADQ, were found.

### Service use

The use of services used in the previous 6 months at 6 and 12 month follow-up are presented in Table 6. At 6 and 12 months the ACT + TAU group used significantly more alcohol day care services than the TAU group and significantly more alcohol outpatient services at 12 months. At 6 months the ACT + TAU group had significantly fewer inpatient days, outpatient visits to non-alcohol-related services and significantly more GP visits. No significant differences were observed between the groups at 12 months.

**Table 6.** Mean service utilization (SE) over previous 6 months at 6 and 12 months by allocation

	Month 6			Month 12		
	ACT + TAU	TAU	*P*-value	ACT+TAU	TAU	*P*-value
Alcohol services						
Day care	26.5 (4.9)	12.8 (7.2)	0.12	29.0 (6.9)	14.2 (5.3)	0.09
Outpatient	7.2 (1.3)	5.5 (1.2)	0.39	6.9 (2.04)	2.0 (0.7)	0.02
Inpatient nights	33.2 (10.4)	39.2 (10.9)	0.7	61.1 (19.3)	44.1 (13.0)	0.5
Other NHS services						
Emergency department	1.3 (0.4)	2.0 (0.9)	0.46	1.1 (0.3)	1.4 (0.5)	0.68
Inpatient nights	1.2 (9.1)	26.8 (6.7)	0.2	1.2 (0.8)	4.2 (3.2)	0.4
Outpatient	1.3 (0.2)	1.9 (0.3)	0.1	2.2 (0.9)	1.6 (0.3)	0.56
Day case	0.6 (0.5)	0	0.4	0.4 (0.4)	0.2 (0.2)	0.63
GP visit	6.3 (0.6)	4.2 (0.7)	0.02	6.2 (0.9)	5.6 (0.8)	0.62

### Discussion

This study demonstrates the feasibility of recruitment and retention of participants in a randomized controlled trial of ACT + TAU versus TAU. Of the 126 patients referred to the research team, 80.3% provided consent and were randomized. This compares favourably with previous RCTs in similar clinical populations where eligibility and consent rates were generally lower (UKATT Research Team, 2005). Further, the follow up rates were higher than in many previous RCTs with similar alcohol dependent clinical populations. We achieved a 92.6% follow-up rate at both 6 months and 12 months, exceeding our minimum target follow up rate of 75% at both follow up points, and may reflect the use of optimal methods to maximize follow-up (Woolard *et al*., 2004; Robinson *et al*., 2007; Zweben *et al*., 2009). The follow up rate was lower in the TAU group compared to the ACT + TAU group, which may have been related to the more intensive nature of the ACT intervention arm, and potentially a degree of disappointment in those randomized to TAU (Woolard *et al*., 2004). However, the difference in follow up rate was within acceptable limits and greater than the planned minimum follow up rate in both groups.

In terms of implementation of the ACT intervention, we found that the clinical teams were able to engage participants for a significantly longer period of time than in TAU, and close to the intended 365 days. There was no evidence of contamination between the two study arms. The ACT group also received a significantly greater number of contacts with specialist staff, and contacts were more often in participants’ homes or local communities, than in the TAU only group. Given that there are considerable differences in assertiveness, intensity and duration of treatment between ACT and treatment typically delivered by addiction services, this study demonstrates the feasibility of implementation of ACT in this population.

This pilot study was not designed to be statistically powered to provide a definitive test of the effectiveness of ACT + TAU versus TAU alone. Nevertheless there were some differences in outcome between the two groups principally at 6 months follow up, with the ACT + TAU reporting fewer drinking days but lower quality of life and greater alcohol-related problems than TAU. The ACT + TAU group also had greater engagement with alcohol services at both 6 and 12 months and significantly less unplanned inpatient care and outpatient hospital visits than the TAU group. The ACT + TAU group also had significantly more GP visits at 6 months. These differences may reflect improved facilitated access to planned health interventions supported by the ACT staff and a beneficial reduction in unplanned care. Overall this pilot RCT demonstrated the feasibility of conducting a trial of ACT + TAU versus TAU. A definitive trial is now warranted.
